# Evaluating homophily in networks via *HONTO* (HOmophily Network TOol): a case study of chromosomal interactions in human PPI networks

**DOI:** 10.1093/bioinformatics/btac763

**Published:** 2022-11-28

**Authors:** Nicola Apollonio, Daniel Blankenberg, Fabio Cumbo, Paolo Giulio Franciosa, Daniele Santoni

**Affiliations:** Institute for Applied Mathematics “Mauro Picone”, National Research Council of Italy, Rome 00185, Italy; Genomic Medicine Institute, Lerner Research Institute, Cleveland Clinic, Cleveland, OH 44195, USA; Genomic Medicine Institute, Lerner Research Institute, Cleveland Clinic, Cleveland, OH 44195, USA; Department of Statistical Science, University of Rome “La Sapienza”, Rome 00185, Italy; Institute for Systems Analysis and Computer Science “Antonio Ruberti”, National Research Council of Italy, Rome 00185, Italy

## Abstract

**Summary:**

It has been observed in different kinds of networks, such as social or biological ones, a typical behavior inspired by the general principle ‘similarity breeds connections’. These networks are defined as homophilic as nodes belonging to the same class preferentially interact with each other. In this work, we present *HONTO* (HOmophily Network TOol), a user-friendly open-source Python3 package designed to evaluate and analyze homophily in complex networks. The tool takes in input from the network along with a partition of its nodes into classes and yields a matrix whose entries are the homophily/heterophily *z*-score values. To complement the analysis, the tool also provides *z*-score values of nodes that do not interact with any other node of the same class. Homophily/heterophily *z*-scores values are presented as a heatmap allowing a visual at-a-glance interpretation of results.

**Availability and implementation:**

Tool’s source code is available at https://github.com/cumbof/honto under the MIT license, installable as a package from PyPI (*pip install honto*) and conda-forge (*conda install -c conda-forge honto*), and has a wrapper for the Galaxy platform available on the official Galaxy ToolShed (Blankenberg *et al.*, 2014) at https://toolshed.g2.bx.psu.edu/view/fabio/honto.

## 1 Introduction

Detecting associations among classes of nodes in a network is a challenging task. In social networks, it has been observed that users sharing the same ethnicity, age or cultural background usually prefer to interact with each other (McPherson *et al.*, 2001). In this case, when interactions between nodes belonging to the same class (intra) occur more often than expected, the network is considered as homophilic while when interactions between nodes belonging to different classes (inter) occur more often than expected the network is considered as heterophilic. In the present work, we developed a tool *HONTO* (**HO**mophily **N**etwork **TO**ol) implementing a method, designed by Apollonio *et al.* (2022), to evaluate homophily/heterophily in networks. The tool was also made available through Galaxy platform (The Galaxy Community, 2022). The core of the method consists in formalizing the concept of *more often than expected* referring to second-order statistics extracted from the considered network itself.

## 2 Statistical background and pipeline

We assume network nodes are partitioned into *s* classes (*colors*), giving a *color profile* $c=(c_{1},\ldots ,c_{s})$ in which *c_i_* is the cardinality of the *i*th color class. For a given graph *G* with *n* nodes, and a given color profile *c*, we consider all possible node colorings *f* having the same color profile *c* as equally likely. This amounts to equip the sample space of node colorings having profile *c* with the uniform distribution $c_{1}!c_{2}!\cdots c_{s}!/n!$. Having the sample space, we consider the $\left(\begin{array}{c} s\\ 2 \end{array}\right)+s$ random variables $M_{i,j}$ defined as the number of *i*, *j*-edges in *G*, i.e. edges whose (random) color of the endpoints lies in the set {*i*, *j*} with possibly *i *=* j*. If i=j, then *i*, *j*-edges are called *heterophilic*, otherwise they are called *homophilic*. Using the results in Apollonio *et al.* (2022), we compute first and second-order moments of the $M_{i,j}$’s. Given the observed coloring $f^{*}$, and hence the observed number $M_{i,j}^{*}$ of *i*, *j*-edges, we compute the *z*-score of each $M_{i,j}^{*}$. Via classical tail inequalities, this *z*-score measures how much the number of observed *i*, *j*-edges in *G*, with possibly *i *=* j*, deviates from its expected value by random chance. For each color *i*, we also consider the random variable *S_i_* defined as the (random) number of nodes of color *i* that have no neighbors of color *i*. We call such nodes *color-i isolated*. Notice that color-*i* isolated nodes are not necessarily isolated in *G*, but they are such in the subgraph induced by the nodes of color *i*. Positive values of *i*, *i*-edge *z*-scores denote homophily (we observe more homophilic edges than expected by random chance), while positive values of *i*, *j*-edge *z*-scores, with i=j, denote heterophily (we observe more heterophilic edges than expected by random chance); the higher the *z*-score the higher the confidence of homophily/heterophily. Finally, negative values of color-*i* isolated *z*-scores also indicates homophily, because, in case of homophily, nodes of a certain color are expected to have neighbors of the same color. Therefore, observing fewer color-*i* isolated nodes than expected supports homophily.

**Fig. 1.**
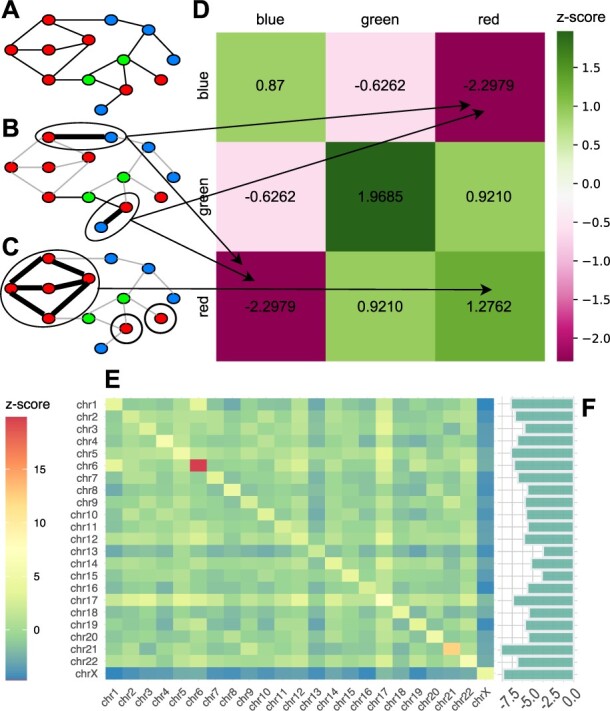
(**A**) a three-colors sample network; the blue, red-edges appear in (**B**), while the subgraph induced by red nodes is shown in (**C**); (**D**) heatmap with the edge *z*-scores computed by *HONTO* on the three-colors sample network reported in (A); (**E**) edge *z*-scores related to *Homo sapiens* PPI network, where proteins are partitioned into chromosomes, are reported along the diagonal for homophily and out of the diagonal for heterophily of heatmap; (**F**) *z*-scores of color-*i* isolated nodes for the *H.sapiens* PPI network (A color version of this figure appears in the online version of this article)

Figure 1 shows a very simple example. The nodes of graph *G* in Figure 1A are colored blue, green and red. The profile of this coloring is (4, 2, 7) which simply means that there are four blue nodes, two green nodes and seven red nodes. We view this coloring as the observed outcome $f^{*}$ from the sample space of all equally likely colorings of *G* of profile (4, 2, 7) (the random coloring model). HONTO takes in input the graph *G* and the coloring $f^{*}$, computes z-scores using the results in Apollonio *et al.* (2022), and outputs the heatmap shown in Figure 1D, obtained by placing in position (*i*, *j*), (i,j)∈{blue,green,red}×{blue,green,red}, the z-score of the number of observed *i*, *j*-edges $M_{i,j}^{*}$ of *G* (possibly *i *=* j*). Just follow the arrows. For instance, the value of the (blue,red)-entry is the z-score of the observed number of blue, red-edges (there are two such edges shown in Figure 1B by thick edges). The graph induced by the red nodes is shown in Figure 1C: it has six edges (thick edges) and two red-isolated nodes. Therefore, there are six red, red-edges with a z-score of 1.2762. Notice that the heatmap is symmetric. The entries of the arrays in Figure 1E and F actually refer to the network we used as a test case (see next section).

## 3 Application example

The human protein–protein interaction (PPI) network was downloaded from the STRING database (Szklarczyk *et al.*, 2021), setting a standard threshold on edge score (*T* = 700). Each protein occurring in the PPI network was assigned to a class corresponding to the chromosome the related gene belongs to. A total of 23 classes (chr1, chr2,…, chr22, chrX) were considered (excluding the class corresponding to chromosome Y because of the small number of genes occurring in the network). The homophily/heterophily nature of the network, with respect to chromosome classes, was evaluated through *HONTO* tool. In other words, the tendency of proteins to preferentially interact with proteins whose genes are physically located on the same chromosome (homophily) or on different chromosomes (heterophily) was investigated. In Figure 1E, *z*-scores related to intra- (along the diagonal) and inter-chromosomal interactions (other than the diagonal) are reported as a heatmap. As one can observe, values on the diagonal are clearly higher than off-diagonal values, leading to assess a homophilic nature of the network, confirming the link between shared chromosome and interaction in the PPI network. This result can complement, to some extent, the study of correlations between the distance, expressed in terms of base pairs, of genes on the same chromosome and the distance of corresponding proteins in the PPI network in *Saccharomyces cerevisiae* (Santoni *et al.*, 2013). In Figure 1F, *z*-scores of color-isolated nodes are presented as a histogram. Coherently with *z*-scores shown in Figure 1E, all *z*-scores of color-isolated nodes are significantly negative, meaning that the number of color-isolated nodes is smaller than expected, confirming the homophilic nature of the network. Instructions on how to reproduce both the heatmap and the histogram, together with the results produced by *HONTO* on the STRING PPI network, are available at https://doi.org/10.5281/zenodo.6941315.

## 4 Conclusion

Here, we presented *HONTO*, a powerful tool for evaluating homophily in complex networks. It is extremely easy to use and domain-agnostic, in the sense that it can be applied on networks independently from their scientific domain. To our knowledge, *HONTO* is the first tool able to provide a quantifiable score, expressed in terms of probability, associated with the homophilic nature of networks.

## Data Availability

Data used in the article is publicly available in the reference item Szklarczyk.
